# An uncommon complication of a common injury: Acute foot compartment syndrome following an ankle sprain: A case report and review of the literature

**DOI:** 10.1097/MD.0000000000035660

**Published:** 2023-10-20

**Authors:** Joeffroy Otayek, Chahine Assi, Kaissar Yammine

**Affiliations:** a Department of Orthopedic Surgery, Lebanese American University Medical Center-Rizk Hospital, Lebanese American University, School of Medicine, Beirut, Lebanon; b Center for Evidence-Based Anatomy, Sport and Orthopedics Research, Beirut, Lebanon; c Foot and Ankle and Diabetic Foot Clinic, Lebanese American University Medical Center-Rizk Hospital, Beirut, Lebanon.

**Keywords:** acute compartment syndrome, ankle sprain, foot compartment syndrome

## Abstract

**Introduction:**

Acute foot compartment syndrome (FCS) is a rare but potentially devastating complication that can occur following severe trauma of the limbs. In very are cases, such syndrome occurs following minor trauma. We present an exceptional case of acute FCS as a complication of an ankle sprain.

**Clinical findings:**

A 32-year-old male patient presented with excruciating foot pain and swelling 48 hours following an ankle sprain. Physical examination revealed severe swelling of the right foot, pale and swollen toes, and tense and pale dorsal skin and severe pain upon passive extension of the toes.

**Diagnosis:**

An acute FCS was considered.

**Intervention and outcomes:**

The patient underwent a fasciotomy using a double-dorsal incision technique. The patient’s symptoms were controlled, and he was discharged from the hospital 2 days after the surgery.

**Conclusion:**

Acute FCS could occur following minor trauma such as an ankle sprain. Early recognition and timely surgical intervention are crucial to prevent severe complications. The diagnosis is primarily clinical and immediate fasciotomy is needed to reduce intracompartment pressure and prevent muscular necrosis and other complications.

## 1. Introduction

Foot compartment syndrome (FCS) is a rare but potentially limb-threatening complication that can occur following trauma to the foot or ankle. FCS occurs when the pressure within a muscle compartment exceeds the perfusion pressure, leading to tissue ischemia and necrosis.^[1]^ The diagnosis of FCS can be challenging, and the decision to perform an emergency fasciotomy is often difficult, particularly in cases where the diagnosis is primarily clinical. This case report presents a young male who developed FCS following a simple ankle sprain and discusses the challenges in its management.

## 2. Case presentation

A previously healthy nonsmoker 24-year-old man presented to our emergency department late at night with excruciating pain in his right foot, accompanied by an inability to walk or bear weight. The patient had a history of a twisting injury to his right ankle during a basketball game 2 days prior to presentation, for which he sought medical attention at another facility. At that time, physical examination revealed tenderness and swelling over the lateral malleolar area, reduced range of motion of the ankle joint, and moderate instability upon anterior drawer testing. Radiographs of the ankle joint were negative for fracture, and he was diagnosed with a 2nd degree lateral ankle sprain and prescribed Rest Ice Compression Elevation protocol. He was advised to avoid weight-bearing and scheduled for a follow-up examination in a week.

**Figure 1.**
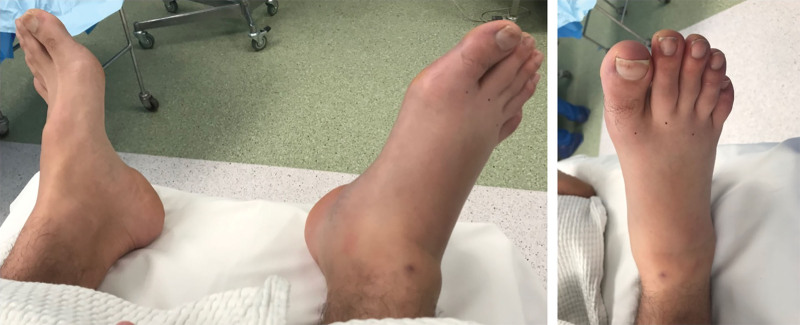
Clinical picture showing severe swelling of the right foot upon presentation to the emergency department.

Upon presentation to our institution, the patient reported that the pain had started 3 hours earlier and had progressively worsened, despite receiving morphine-like pain medications. Physical examination revealed severe swelling of the right foot, reduced range of active and passive motion, pale and swollen toes, and tense and pale skin (Fig. 1). There was pain at the dorsum of the foot upon passive extension of the toes. The patient’s vital signs showed a temperature of 36.5 degrees Celsius, pulse rate of 118 beats per minute, and blood pressure of 130/80. Blood test results were all normal. The patient’s pain continued to worsen despite morphine administration.

**Figure 2.**
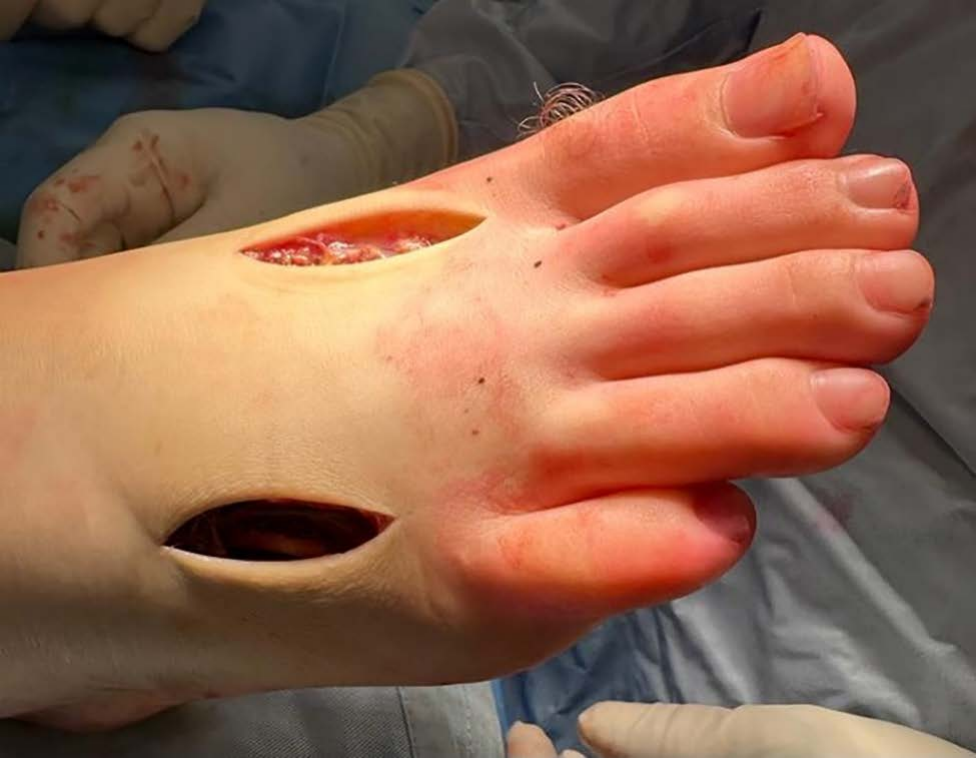
Dorsal dual incision used for urgent fasciotomy.

The dorsalis pedis and posterior tibial pulses were not palpable and were not detectable on Doppler ultrasound. Radiographs were negative for fracture, and no signs of deep vein thrombosis in the left leg were noticed. A diagnosis of FCS was suspected, and the patient was taken emergently to the operating room within 5 hours of presentation. Emergency fasciotomy of the lateral and medial compartments of the foot was performed through dual dorsal incisions, overlaying the second and 4th metatarsals (Fig. 2). All foot web spaces and compartments were carefully opened, and necrotic muscle tissue was excised. Within minutes of surgery, the skin color turned from pale to pink. The wounds were left open, and the patient was placed in a posterior splint.

**Figure 3.**
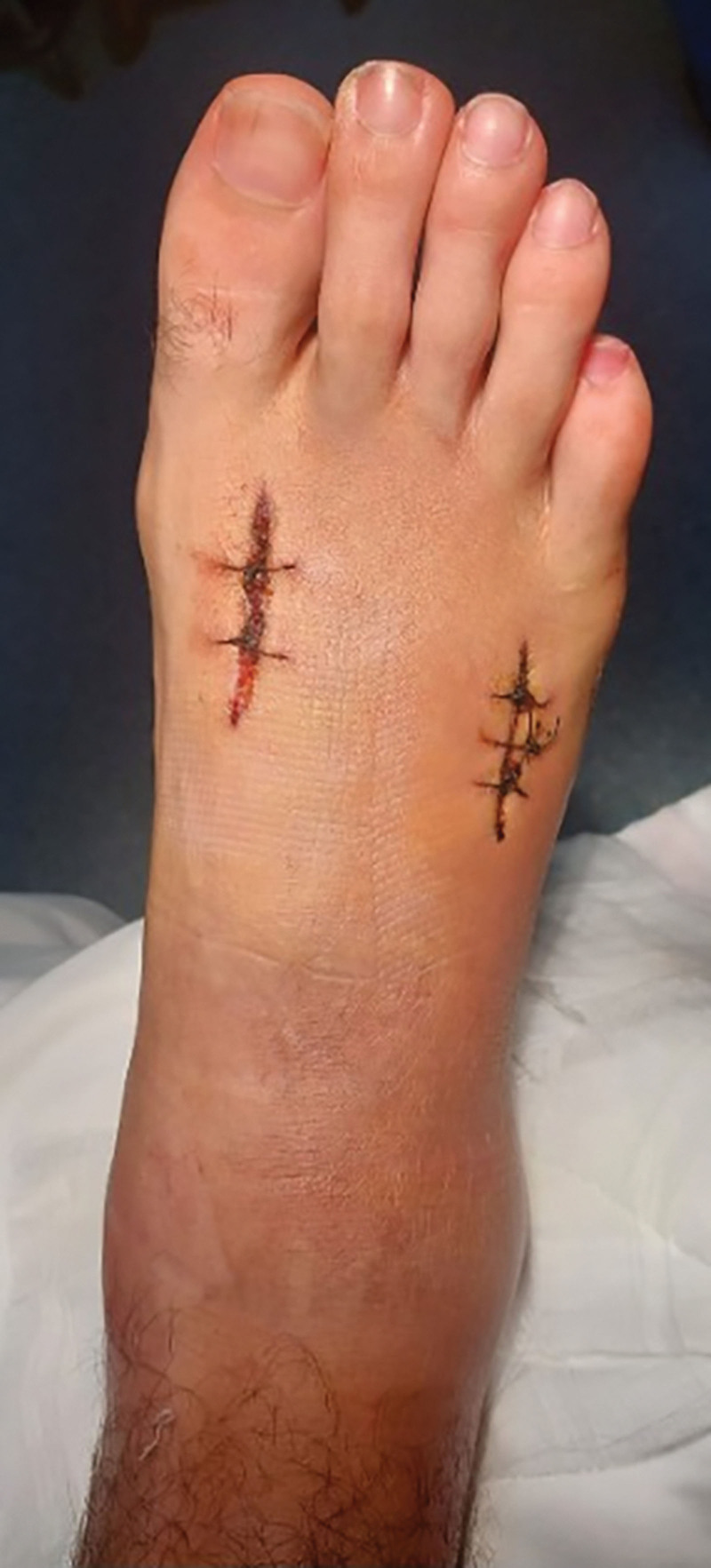
Direct closure of the wounds three weeks after the fasciotomy.

Following the surgery, the patient’s symptoms improved dramatically, and he was discharged from the hospital 2 days later, with instructions for non-weight-bearing for 20 days and a below-knee posterior plaster splint. The patient followed rehabilitation exercises at home for 2 months. Direct closure of the wounds was performed 3 weeks after the fasciotomy without the need of skin graft (Fig. 3). The patient made a full recovery, with full function of his foot, including full range of motion of his right ankle and foot. There were no contractures of the toes or deformities of the affected leg or foot. He remained pain-free throughout his recovery.

Ethical approval from the Institutional Review Board has been waived since the study is retrospective and comprises <3 subjects.

## 3. Discussion

This case report presents a rare instance of an acute FCS developing as a complication of a “benign’’ second-degree ankle sprain, without any associated fractures detected on radiographs imaging. Acute compartment syndrome (ACS) typically occurs following major soft tissue injury, vascular trauma, or fracture.^[1]^ The foot is a relatively uncommon site for this condition, with a prevalence of only 5%.^[2]^ If left untreated, ACS can result in irreversible nerve injuries, muscle necrosis, ischemic contractures, or tissue necrosis, ultimately necessitating amputation.^[3]^ Thus, a high level of clinical suspicion and prompt diagnosis are crucial for successful management of this condition. In this particular case, intracompartment pressure measurement was not available in our institution, and diagnosis was made primarily through clinical evaluation.

The clinical diagnosis of ACS is primarily based on a combination of physical signs and symptoms. These include increased pain, which is disproportionate to the original injury, along with pallor, paresthesia, paralysis, and pressure within the affected compartment.^[3,4]^ Patients with FCS may experience overt pain, which is further exacerbated by active and passive range of motion of the ankle joint, forefoot, or toes. According to Myerson et al^[5]^ retrospective study of 14 patients treated for compartment syndrome after foot injury, pain on passive dorsiflexion of the toes was found to be the most reliable clinical finding for distinguishing the pain of an impending compartment syndrome from that of the initial injury. Additionally, they may have sensory deficits specific to the affected compartment and a significant amount of swelling. While pulselessness may be present in the late stages of ACS, it is not a common finding. The examination of pulses is an unreliable indicator of ACS, as the intracompartment pressure may not reach systolic pressure. It is important to note that necrosis of the soft tissues of the foot may occur even with compartment pressures ranging from 30 to 60 mm Hg.^[6]^

To the best of our knowledge, 6 cases of acute FCS following an ankle sprain were identified in the literature. These are presented in Table 1.

**Table 1:** Case reports of acute foot compartment syndrome following an ankle sprain.

Study	Context of foot compartment syndrome	Association with vascular injury	Treatment
Kym et al^[7]^	Pseudoaneurysm of dorsalis pedis artery	Yes	Surgical decompression, artery repair
Dhawan et al^[8]^	Disruption of anterior tibial artery	Yes	Fasciotomy
Creighton et al^[9]^	Recurrent ankle inversion injury	No	Not mentioned
Cortina et al^[10]^	Deltoid ligament rupture	No	Fasciotomy
Maurel et al^[11]^	Minor ankle sprain	No	Three-incision decompression
Christoforidis et al^[12]^	Grade II ankle sprain	No	Emergency fasciotomy, muscle removal

Limited research has been conducted on the management and prognosis of lower extremity and FCS that goes undiagnosed initially, with only a handful of studies published on the topic.^[7]^ Rosenthal et al^[7]^ have reported long-term consequences such as permanent loss of function, contracture, painful warts, toe clawing, and sensory disturbances, and therefore advocate for early and comprehensive surgical intervention.

Fasciotomy is the definitive treatment for compartment syndrome and must be performed emergently to allow venous circulation to recover.^[8]^ Several approaches are described for FCS, including plantar approach, dorsal approach, medial-plantar approach, and lateral.^[9]^ The double incision dorsal approach used in this case allowed the surgeon to gain access to almost all 9 compartments of the foot. For the medial approach, it is recommended to incise and dissect medial to the second metatarsal, and for the lateral compartments, the incision should be made lateral to the 4th metatarsal. Proper spacing between the 2 dorsal incisions is crucial to reduce the risk of tissue necrosis. This approach is highly effective and allows for appropriate surgical management of foot compartment syndrome.

Although of limited design, this case report highlights the importance of the clinical examination even when dealing with a “minor’’ injury. Measurement of the intra-compartmental pressure could be considered as another limitation. However, the primacy of positive signs and symptoms along with the non-responsiveness of pain to morphine medication should be sufficient for the diagnosis with no treatment delay.

## 4. Conclusion

In conclusion, foot compartment syndrome is a rare but potentially devastating complication that can occur even after a seemingly minor injury. Early recognition and intervention are crucial for preventing irreversible damage to the tissues and avoiding the need for amputation. In our case, the diagnosis was primarily clinical due to the unavailability of intracompartment pressure measuring devices in our institution. The double incision approach for fasciotomy enabled the access to all 9 compartments of the foot. Despite the challenges in managing such cases and the difficulty in deciding to operate, surgical intervention was successful in preventing further damage to the tissues and preserving the patient’s foot function.

## Acknowledgments

We also thank the healthcare professionals who were involved in the patient’s care. Finally, we extend our appreciation to our colleagues for their feedback and support in the preparation of this report.

## Author contributions

**Conceptualization:** Joeffroy Otayek, Kaissar Yammine.

**Formal analysis:** Kaissar Yammine.

**Methodology:** Joeffroy Otayek.

**Investigation:** Chahine Assi.

**Supervision:** Kaissar Yammine.

**Writing – original draft:** Joeffroy Otayek, Chahine Assi, Kaissar Yammine.

**Writing – review & editing:** Joeffroy Otayek, Chahine Assi, Kaissar Yammine.

## References

[1] Dodd A Le I. Foot compartment syndrome: diagnosis and management. J Am Acad Orthop Surg. 2013;21:657–64. 24187035 10.5435/JAAOS-21-11-657

[2] Thakur NA McDonnell M Got CJ. Injury patterns causing isolated foot compartment syndrome. J Bone Joint Surg Am. 2012;94:1030–5. 22637209 10.2106/JBJS.J.02000

[3] Malik AA Khan WSA Chaudhry A. Acute compartment syndrome – a life and limb threatening surgical emergency. J Perioper Pract. 2009;19:137–42. 19517954 10.1177/175045890901900503

[4] Frink M Hildebrand F Krettek C. Compartment syndrome of the lower leg and foot. Clinical Orthopaedics and Related Research. Springer New York LLC; 2010. 10.1007/s11999-009-0891-x PMC2835588 19472025

[5] Myerson MS. Management of compartment syndromes of the foot. Clin Orthop Relat Res. 1991:239–48. 1680591

[6] Elliott KGB Johnstone AJ. Diagnosing acute compartment syndrome. J Bone Joint Surg Br. 2003;85:625–32. 12892179

[7] Rosenthal R Tenenbaum S Thien R. Sequelae of underdiagnosed foot compartment syndrome after calcaneal fractures. J Foot Ankle Surg. 2013;52:158–61. 23321291 10.1053/j.jfas.2012.11.016

[8] Bedigrew KM Stinner DJ Kragh JF. Effectiveness of foot fasciotomies in foot and ankle trauma. J R Army Med Corps. 2017;163:324–8. 28341786 10.1136/jramc-2016-000734

[9] Lutter C Schöffl V Hotfiel T. Compartment syndrome of the foot: an evidence-based review. J Foot Ankle Surg. 2019;58:632–40. 31256897 10.1053/j.jfas.2018.12.026

